# Rocks Ahead

**Published:** 1889-04-20

**Authors:** 


### The Hospital

A Weekly Institutional Journal of

**Science, Medicine, Nursing, and Philanthropy.**

*For Hospitals, Asylums, Medical Practitioners, Students, Nurses, Families, Parishes, Congregations, and the Charitable Public.*

Vol. VI. No. 134.] [April 20, 1889.

### "Rocks Ahead."

If Mr. W. R. Greg were alive he would pardon the temporary use of the title of his famous book. The ship of medical charity has got a little out of hand during the last fifteen or twenty years, and we are drifting into unknown seas, where charts and light-houses are of little avail. "Drifting" is not a proceeding which commends itself to men of capacity if it can be avoided. Even in the worst of circumstances there should still be a man at the helm and a captain ready to give orders at the favourable moment. The water at the present time is comparatively smooth and the sky serene; but, undoubtedly, there are dangerous "rocks ahead" in the direction in which the ship is driving, and these it is everybody's business to keep her clear of.

Medical charity, to change the figure, having been a flower, is threatening to degenerate into a weed, a quick-growing, crop-choking, dangerous weed. The proportion of the population brought within its influence year by year is steadily increasing. Instead of being a beneficent resource for the extremely needy in the hour of their stern distress, it is rapidly becoming a realisable possession for all who choose to claim it, whether needy or not. It does not require more than a moment's consideration to understand that that is an injury and a degradation to all claimants who are not necessitous. The first step in pauperism, like the first step in drunkenness or crime, is always the most difficult to take. When once taken, it is with the new convert to the pauper faith as with the new convert to drunkenness, he outruns his older associates in the eagerness of his zeal. *Facilis decensus Averni.*

The movement which has been recently set on foot with such commendable energy by the Hospital Saturday Fund, with the Lord Mayor at its head, is in its inception and its motives entirely and absolutely good. That the working class should wish to support hospitals, and that hospital managers should encourage their enthusiasm, is the most natural thing in the world. But whither does the movement tend, where does it lead to? Can it for a moment be supposed that the working class when they have raised their hundred thousand a year will hand it over as a free gift to the present managers of hospitals without question and without stipulation? We know very well they will not. The working men understand as well as any other class that taxation and representation in this country have always been twin sisters. The principles involved are the same whether the taxation is voluntary or compulsory. The working man knows well the meaning of a *quid pro quo*. If he gives a hundred thousand a year to hospitals, he intends not only to decide what particular charities shall share in his liberality, but also to help in the spending of the money at each institution. Can he be blamed for that? Assuredly not.

But the rock which lies ahead here is this: the working man may begin to think that because he contributes his penny a week and his hundred thousand a year to hospitals, therefore he is paying for what he gets, and is no longer a recipient of charity. Not a recipient of charity! If a starving traveller pays a penny for a loaf the real value of which is a shilling, whilst the other eleven pence are contributed by the benevolence of the wealthy, is he not a recipient of charity? If the working man should ever come to regard hospitals as his just possessions because he contributes a fiftieth part of their cost, his moral sense will be more injured than if he paid nothing at all. When he accepts medical relief for which he has paid nothing, he merely takes one step in the direction of pauperism; but when he accepts relief *as his just right* for which he has paid no more than a strictly limited proportion of the money value, he takes a step in the direction of fraud as well as of pauperism. In accepting free relief as a gift, he merely blunts his finer sense of manhood; in accepting as a right the relief for which he has only paid a part of the value, he imposes a deliberate fraud upon his conscience. If anybody considers that that is a small matter which need not concern practical men, he has yet to learn that a bad principle which has obtained the general sanction of the world is much more injurious to the community than the vilest imaginable practices which are the objects of universal reprobation.

Do we, then, desire to stand in the way of either workmen's contributions, or the representation of workmen on hospital boards of management? By no means. But we do desire some limitation of the extraordinary floods of more or less free medical relief which are overflowing the land as with a devastating deluge. The methods of relief which are available for the poor may be broadly divided into two classes: poor-law infirmaries and charitably maintained hospitals. As they are at present conducted, there is no strict line of demarcation between the two. For all practical purposes they are both supported by one and the same class, and that class is entirely distinct from those by whom they are used. The general hospitals are as much maintained by the money of the wealthy as are the poor-law infirmaries. The only real difference is that in the former case the wealthy tax themselves, whereas in the latter they are taxed by the State. To hold, therefore, that a man is degraded to the level of pauperism if he goes to a poor-law infirmary, whilst he retains his full self-respect and independence if he goes to a general hospital, is sheer nonsense. The whole question of medical relief of the poor by means of voluntary and State aid is in a condition of the most hopeless and unmitigated chaos. The consequences are that beggars in the garb of hospital supporters and managers are multiplied by the thousand, whilst nobody can put upon them the least check of any kind. The rich are milked like helpless cows, and the medical practitioners are ruined by utterly unscrupulous competition.

Many people will admit and deplore the present disgraceful and ruinous muddle; but they are quite hopeless as to the prospect of any change for the better. We do not agree with them for a moment. Let the daylight be persistently poured in upon the facts, and slowly, but surely, all thoughtful people will become ashamed of them. Shame is the beginning of amendment. But the prospects of change for the better are by no means so remote as many people imagine. The working classes have begun to recognise that it is their duty to do something towards providing for their own medical relief. All that is wanted is that they shall push their present views to their logical issue, and then they will see clearly that it is their duty not only to do something towards providing medical attendance for themselves, but to do everything. That ought to be the final issue of the movement which has just been commenced. Those workmen who are in full work ought no more to demand help in time of sickness, except what is of their own providing, than they ought to demand food, clothing, or house rent. The principle of combination which has established trades unions and maintained benefit societies is amply sufficient for the provision of the best medical relief. If it be said that many working men are not in full or in well-paid work, and that they cannot provide medical relief for themselves, the answer is obvious—What are the costly and excellent poor-law infirmaries for? They are surely for the very class who cannot adequately help themselves.

There are those who will see difficulties in the fact that we have already in operation an established hospital system which is supported by voluntary contributions, and that this system cannot be replaced all at once by the self-helping system which working men should seek to establish. But there is no necessity for replacing it. The working men should combine and, by the steady increase of their resources, gradually, and little by little, take over the existing voluntary hospitals. They should support them entirely, and then they would be entitled to manage them entirely. At present the hospitals are managed solely for working men; they would, if taken over, be managed solely by them as well as for them. The existing managers, who devote so much time and money to hospitals, would be most thankful to be relieved of their burdens, and to turn their attention to other pressing philanthropies. Of course, there would be, for a time, some dislocation of the arrangements which obtain with regard to medical schools. But the medical school difficulty would soon be got over. Instead of the voluntary hospitals being the sole or the principal places of medical education, the honours would in future be with the poor-law infirmaries. It is impossible to compute the manifold and invaluable results which would arise to the community and to the medical profession if the prosperous portion of the working classes would accept the full responsibility of providing for themselves when sick. The blot upon our civilisation, of crowded out-patient departments and of a pauperised industrial class, would be wiped out: the scandals of hospital begging and hospital extravagance would cease; the starving medical man would see his family again provided with bread; and the lion of the consulting-room would lie down with the lamb of general practice. May the gods grant it!

